# Adolescent Alcohol and Stress Exposure Rewires Key Cortical Neurocircuitry

**DOI:** 10.3389/fnins.2022.896880

**Published:** 2022-05-17

**Authors:** Avery R. Sicher, Arielle Duerr, William D. Starnes, Nicole A. Crowley

**Affiliations:** ^1^The Pennsylvania State University, University Park, PA, United States; ^2^Department of Biology, The Pennsylvania State University, University Park, PA, United States; ^3^Department of Biomedical Engineering, The Pennsylvania State University, University Park, PA, United States

**Keywords:** alcohol, stress, prefrontal, neurocircuitry, development, adolescence

## Abstract

Human adolescence is a period of development characterized by wide ranging emotions and behavioral risk taking, including binge drinking (Konrad et al., 2013). These behavioral manifestations of adolescence are complemented by growth in the neuroarchitecture of the brain, including synaptic pruning (Spear, 2013) and increases in overall white matter volume (Perrin et al., 2008). During this period of profound physiological maturation, the adolescent brain has a unique vulnerability to negative perturbations. Alcohol consumption and stress exposure, both of which are heightened during adolescence, can individually and synergistically alter these neurodevelopmental trajectories in positive and negative ways (conferring both resiliency and susceptibility) and influence already changing neurotransmitter systems and circuits. Importantly, the literature is rapidly changing and evolving in our understanding of basal sex differences in the brain, as well as the interaction between biological sex and life experiences. The animal literature provides the distinctive opportunity to explore sex-specific stress- and alcohol- induced changes in neurocircuits on a relatively rapid time scale. In addition, animal models allow for the investigation of individual neurons and signaling molecules otherwise inaccessible in the human brain. Here, we review the human and rodent literature with a focus on cortical development, neurotransmitters, peptides, and steroids, to characterize the field’s current understanding of the interaction between adolescence, biological sex, and exposure to stress and alcohol.

## Introduction

### Adolescence Is a Key Window of Neurodevelopment

Adolescence is defined as the transitional period between childhood and adulthood marked by biological, behavioral, and cognitive changes (Spear, 2000). Human adolescents show heightened risk taking and novelty seeking behavior, peaking in early adolescence, and then decreasing throughout late adolescence and early adulthood (Steinberg et al., 2008), possibly contributing to high rates of drug use during this period (Spear, 2000). Adolescence is also a period of intense brain maturational changes. Among brain structures, the prefrontal cortex (PFC) is one of the last to mature, potentially making the PFC particularly vulnerable to changes induced by things like stress and alcohol use – the exposure to which are already heightened during this time (Larsen and Luna, 2018).

The purpose of this review is to outline major neurological and behavioral changes which occur during adolescence and describe how exposure to stress or alcohol (or combined exposure) during adolescence can interfere with these developmental processes. We focus primarily on changes occurring in the PFC, as it is one of the final brain regions to fully mature and is known to be altered by alcohol consumption or exposure. In addition, the medial region of the PFC has been well connected to alcohol-seeking behaviors (for review, see Klenowski, 2018). In this review, we will highlight major developmental changes in the PFC including myelination, synaptic pruning, and neurotransmitter and neuropeptide systems, that are vulnerable to adolescent stress and alcohol. For the purposes of this review, biological sex is recapitulated as captured in the original manuscripts.

Human adolescence is estimated to comprise most of the second decade of life (human ages 10–20), although some argue that biological and social factors point toward a broader window spanning ages 10–24 (Sawyer et al., 2018). Adolescents become more social and seek to interact with their peers more during this time (Spear, 2000). Other adolescent-typical behavioral changes include risk-taking, intense emotions, and sensation-seeking (Spear, 2000; Konrad et al., 2013). Risk-taking behavior can have consequences including substance use, car accidents, and unsafe intercourse (CDC, 2020).

Animal models can be used to study and characterize many of the neurobiological changes associated with adolescence which cannot be feasibly studied in humans. Rodent animal models show a transitional period between juvenile and adulthood stages marked by physical growth, final neurobiological maturation, and behavioral characteristics like those seen in humans (Spear, 2000). As with humans, the exact ages delineating adolescence in rodents are debated. The most conservative definitions of the adolescent period use postnatal day (PND) 28–42 (e.g., Spear, 2000); however, others have identified a wider age range ending around PND 65, arguing that maturational changes continue through this time (Tarazi and Baldessarini, 2000; Spear, 2015). Behaviors characteristic of human adolescence are also present in animals, including changes in social behavior and risk-taking (for review see Spear, 2000). Here, we highlight some of the major changes observed in the PFC during rodent and human adolescence, including synaptic pruning, myelination, and maturation of key neurotransmitter systems, which are altered by adolescent alcohol use.

## Brain Architecture During Adolescence

A major characteristic of adolescent brain development is the remodeling and growth of gray and white matter. Cortical gray matter volume changes according to an inverted U-shape over the lifespan, with increases throughout childhood, a peak in adolescence, and a steady decline throughout adulthood (Giedd et al., 1999). Synaptic pruning, the process by which neuronal communication is refined when excess connections between neurons are eliminated, occurs in the frontal lobe into early adolescence in humans (Huttenlocher, 1979) as well as after the onset of puberty in male and female rodents, suggesting a role for gonadal hormones in this process (Drzewiecki et al., 2016). Unsurprisingly, much of our understanding of the cellular level changes seen in the brain’s architecture during development comes from the animal literature. Neuronal cell loss in ventral regions of the PFC may contribute to gray matter reductions during adolescence, and this effect is much more pronounced in females (Markham et al., 2007). By adulthood, males have more neurons and glial cells in the PFC (Markham et al., 2007; Koss et al., 2015). Pre-pubertal ovariectomy prevented the typical adulthood reduction of neurons and glial in the cortex of females, but castration in males did not alter the number of neurons and glial, suggesting a role of ovarian hormones in neuronal loss (Koss et al., 2015). At the same time, white matter in the PFC increases during adolescence because of myelination in both humans (Miller et al., 2012) and rats (McDougall et al., 2018). Myelination of PFC fibers and synaptic pruning are associated with improvements in inhibitory control of behavior and improved cognition (Caballero et al., 2020) suggesting this process of synaptic refinement is key for behavioral development.

Along with structural development in the PFC during adolescence, innervation of the PFC by several neurotransmitter systems also develops, including the gamma-aminobutyric acid (GABA) system. Inhibitory inputs onto excitatory pyramidal neurons gradually increase throughout adolescence (Cass et al., 2014). The number of parvalbumin (PV)-expressing neurons, the largest subpopulation of GABAergic neurons (Kubota et al., 1994), increases in the mouse PFC during adolescence (Caballero et al., 2014; Ueda et al., 2015; Du et al., 2018). This upregulation is critical for maintenance of a proper excitatory-inhibitory balance and for processing of information from other regions including the hippocampus (Caballero et al., 2020). Although membrane characteristics and electrophysiological properties of PV-expressing neurons are thought to remain consistent throughout adolescence, excitatory input onto PV-expressing neurons was found to increase during late adolescence (Caballero et al., 2014). Calretinin-expressing GABAergic neurons show the opposite trajectory, showing a decrease throughout adolescence (Caballero et al., 2014; Du et al., 2018). Other populations of GABAergic neurons, including somatostatin (SST)-expressing and vasoactive intestinal peptide (VIP)-expressing interneurons were not found to change in number during the adolescent period (Ueda et al., 2015). However, other studies show an increase followed by pruning of SST-expressing GABAergic cells in females (Du et al., 2018) and their broad role in the PFC functioning (Dao et al., 2020, 2021) warrant further developmental investigation. There are also developmental changes in the interaction between other neurotransmitter systems and PFC GABA neurons. Stimulating dopamine receptor 2 (D2Rs) on PFC GABA neurons can increase the firing of these interneurons only in postpubertal rats (Tseng and O’Donnell, 2007), suggesting a development shift in either the function or expression of D2Rs. Glutamate *N*-methyl-D-aspartate (NMDA) receptors are also necessary for development of this GABAergic system and for the proper excitatory/inhibitory balance in the PFC, indicating a role of glutamatergic input (Thomases et al., 2013; Flores-Barrera et al., 2020). These results indicate that proper development of the GABAergic PFC system is necessary for the correct excitatory/inhibitory balance and contributes to processing of afferent input in the adult PFC.

Glutamatergic connections in the PFC finish developing during adolescence. Glutamate binding to NMDA receptors in the frontal cortex increases until PND 28 and declines into adulthood, which could be indicative of changes in NMDA receptor expression or receptor sensitivity throughout the lifespan (Insel et al., 1990). Prior to the adolescent period, there is a shift in NMDA receptor, with NR2A subunits increasing in expression and a corresponding decrease in NR2B subunits (Ueda et al., 2015). However, overall functional maturity of the PFC glutamatergic system is not achieved until later in adolescence (Ueda et al., 2015). The relative function and expression of NMDA receptor subunits changes throughout adolescence. For example, despite the reported decline in NR2B expression during juvenile stages (Ueda et al., 2015), input-specific NR2B function specifically does not develop in the PFC until late adolescence (Flores-Barrera et al., 2014). This indicates that the glutamatergic system of the PFC undergoes profound and complex developmental changes which warrant further in-depth investigation.

In addition to previously mentioned changes in dopamine-mediated input onto PFC GABA neurons, the dopaminergic system itself undergoes vast maturational changes in adolescence. Expression of dopaminergic receptors D1, D2, and D4 continues to increase until PND 60 (Tarazi and Baldessarini, 2000). However, other studies show that dopaminergic receptor expression is lower in early adulthood (PND 70) than in mid-to-late adolescence (PND 45) (Naneix et al., 2012). While other timepoints were not investigated in this study, this suggests that like other neurotransmitter and receptor systems, the dopaminergic system may have a U-shaped developmental trajectory. In humans, levels of mRNA for dopamine receptor 1 (D1R) peak in adolescence before declining throughout adulthood, while D2R mRNA levels decrease after infancy (Weickert et al., 2007). At the same time, dopaminergic axons arising from non-cortical regions, such as the ventral tegmental area and periaqueductal gray, and corresponding dopamine levels increase throughout adolescence (Naneix et al., 2012). These dopaminergic axons continue to grow into the medial PFC and guide the maturation of dendritic spines on medial PFC pyramidal neurons (Reynolds et al., 2018).

The relatively late maturation of the PFC compared to other brain regions has been hypothesized to contribute to risk-taking and sensation-seeking characteristics of adolescents. In contrast to the PFC, other reward-associated structures including the dorsal striatum and nucleus accumbens mature earlier in adolescence [for review of this model, see (Steinberg, 2008)]. This maturational imbalance has been hypothesized to contribute to adolescent-typical risk-taking (Casey et al., 2010). The late-occurring developmental changes in the PFC throughout adolescence likely make the PFC particularly vulnerable to the perturbations caused by stress and alcohol use during this time, as compared to adulthood exposures. Importantly, this also suggests that insults to PFC maturation may induce lifelong consequences in behavior and brain structure. This makes it critical to understand the ramifications of adolescent stress exposure and alcohol consumption, as well as their prevalence and consequences, both from a prevention and intervention standpoint and to identify specific neuronal vulnerabilities.

## Human Studies of Alcohol Exposure

A breadth of literature suggests that human adolescents are at an increased risk of substance use and misuse. According to the 2020 *Monitoring the Future Study*, 25% of 8th graders have used alcohol at least once, increasing to 46% of 10th graders and over 60% of 12th graders – all of which are under the legal drinking age in the United States (CDC, 2020; Miech et al., 2021). The National Institute on Alcohol Abuse and Alcoholism (NIAAA) reports that individuals aged 14–20 are more likely to binge drink (defined by the NIAAA as consuming 5 or more alcoholic drinks for men or 4 or more alcoholic drinks for women, typically within a 2 h period and reaching a blood alcohol concentration over 80 mg/dL), with over 4.2 million individuals aged 14–20 reporting having engaged in binge drinking at least once in the past month in 2019. It is also important to note that these thresholds of ‘binge drinking’ were defined with the adult physiology in mind, and it will be important to consider that these parameters of alcohol consumption are likely to have more severe consequences in the developing human body. Supporting this, recent studies estimate that adolescents, especially those younger than 14 years old, may reach blood alcohol concentrations exceeding 80 mg/dL with as few as 3 alcoholic drinks in adolescent girls and 4 alcoholic drinks in adolescent boys (Donovan, 2009). The NIAAA also reports that there has been a recent decrease in adolescent alcohol consumption in boys, while no such decrease has occurred in girls (National Institute on Alcohol Abuse and Alcoholism, 2019) – though updated numbers following the stress and isolation of the COVID-19 pandemic are not yet available. Adolescent girls ages 16–17 constitute the highest risk group for adolescent binge drinking, with an 11.2% prevalence (National Institute on Alcohol Abuse and Alcoholism, 2019). Importantly, binge drinking often precludes later development of an alcohol use disorder (AUD) (Deas, 2006). The decrease in alcohol use among adolescent men, but not adolescent women, suggests that the gap between AUD prevalence in men and women will continue to narrow (Grant et al., 2015). These rapidly evolving public health statistics show the importance of including males and females in research in preclinical animal models, as well as separating clinical adolescent data by biological sex.

Risks of adolescent alcohol consumption include those which cause immediate bodily harm such as alcohol poisoning and car accidents (individuals aged 16–17 are 17 times more likely to die while in a car crash if their blood alcohol is above 0.08%) (Center for Disease Control, 2012). Adolescent alcohol consumption can also lead to increased events of other risk-related behaviors. Adolescents who have had a binge drinking episode in the last month are more likely to report suicidal ideation than their peers (Duncan et al., 1997; Miller et al., 2007), as well as report behaviors such as engaging in a physical fight, having been a victim of dating violence or sexual assault, and having used other substances (e.g., cocaine, tobacco, and marijuana) (Miller et al., 2007). It will be important to tease apart specific causality for all these other risky behaviors, and their relationship to binge drinking.

Along with these acute risks, adolescent binge drinking is hypothesized to lead to lifelong consequences, partially due to alterations in brain regions including the PFC (Crews et al., 2007). Alcohol consumption early in adolescence, particularly before the age of 15, is associated with increased risk of an AUD later in life (Grant and Dawson, 1997; DeWit et al., 2000). However, other studies suggest early drinking and adulthood AUD may both arise due to familial and environmental factors (Hingson et al., 2006). Causal relationships cannot be conclusively determined from these studies, so identifying potential neurological mechanisms which may increase the risk of adulthood alcohol abuse is important. Longitudinal studies may help identify risk factors for excessive alcohol use and AUD before problematic alcohol use begins.

Neuroimaging studies have found reductions in PFC volume in adolescents with AUD (de Bellis et al., 2005), especially in women (Medina et al., 2008). Interestingly, Medina et al. (2008) demonstrated an interaction between biological sex and AUD diagnosis, as adolescent men with an AUD had larger PFC volumes than adolescent men not diagnosed with an AUD, but adolescent women with an AUD showed reduced PFC volume compared to women without an AUD. In another study focusing on gross cortical changes, Squeglia et al. (2012) reported adolescent women who recently engaged in binge drinking had thicker cortices than their non-drinking counterparts, while adolescent men had thinner cortices compared to their non-drinking counterparts. These results indicate that PFC volume is affected in a sex-specific manner, and that there are differences in consequences between episodes of binge drinking and the progression to AUD. Normal cortical thinning patterns throughout adolescence show different rates of cortical thinning across region and during various timepoints of adolescence (Fuhrmann et al., 2022), suggesting that the age at which an individual begins binge drinking may have differing effects on cortical development. Fuhrmann et al. (2022) identified the midpoint of cortical thinning (MCT) (i.e., the point at which the cortex thins fastest) to be around 15 years of age, with more frontal regions (such as the frontal gyrus) reaching MCT earlier (around age 14) while cingulate regions reached MCT later (around age 17). The cortical alterations seen in these studies may be representative of differing cortical changes across the development of AUD in the two sexes and highlights the need for precise reporting and tracking in cortical subregions.

Alterations in white matter microstructure during early adolescence have been associated with familial history of alcohol use and future instances of binge drinking (Jones and Nagel, 2019). Adolescents who went on to engage in binge drinking tended to have excessive maturation, indicated by increased fractional anisotropy, of tracts connecting striatal regions to the frontal cortex, but insufficient maturation of frontal lobe regions (Jones and Nagel, 2019). However, this study was not sufficiently powered to compare differences between sexes.

Retrospective analyses conducted during adulthood may also provide clarity on cortical changes. An examination of cortical thickness and drinking patterns of United States veterans conducted after September 11, 2001, revealed that individuals with early onset binge drinking (defined as having first binge drinking episode before age 15) had thicker PFCs than post-9/11 veterans who started binge drinking in later adolescence (defined as first binge drinking episode at age 15 or later), and the early onset binge drinkers were at a higher risk for later development of AUD as well as other mental illnesses, as well as having poorer performance on attention and inhibition tests (Bedi et al., 2021). Both groups had thicker PFCs than an age-matched reference group of social drinkers. This suggests a complex interaction between age of onset of binge drinking, stress exposure, cortical thickness, and likely other cortical-related neuropsychiatric disorders such as post-traumatic stress disorder (PTSD) (Jones and Nagel, 2019).

As shown in Bedi et al. (2021), adolescent alcohol consumption also poses concern for present and future psychological illness. Several longitudinal studies demonstrate that drinking alcohol in early adolescence predicts higher risk of alcohol use in late adolescence and early adulthood (Duncan et al., 1997; Enstad et al., 2019; Magee and Connell, 2021). Magee and Connell (2021) report that alcohol consumption at age 17 indirectly predicts higher incidences of Major Depressive Disorder (MDD) in later adulthood (ages 28–30) via two pathways; alcohol use at age 17 was found to predict a continued alcohol use at age 22, which in turn predicted a higher risk for substance use at age 23, leading to a higher risk of MDD at age 30. A similar pathway demonstrated alcohol use at 17 predicting greater depressive symptoms at 22, predicting the same prospects of depressive symptoms at age 23, leading to a higher incidence of a diagnosis of MDD by age 30 (Magee and Connell, 2021). An earlier longitudinal study by Duncan et al. (1997) showed adolescents with a faster rate of acceleration in alcohol consumption across the adolescent period (as compared to peers with a low or steady escalation of alcohol consumption) were more likely to engage in chronic alcohol use in early adulthood and were more likely to experience suicidal ideation in later adolescence. A similar study examining biological sex effects on adolescent drinking found that adolescent boys with a higher drinking frequency were more likely to meet criteria for MDD in late adolescence (by age 17), while adolescent girls with a higher drinking frequency were more likely to meet diagnostic criteria for MDD and comorbid anxiety disorder (Edwards et al., 2014). This sex difference suggests a link between anxiety and adolescent alcohol consumption which may be stronger in women than men. Importantly, it should not be suggested that alcohol consumption definitively leads to increased anxiety, as the relationship between adolescent alcohol consumption and anxiety is unclear beyond a positive correlation.

Though the human literature provides clear evidence for the association between adolescent alcohol consumption, stress, changes in cortical development, and exacerbated problems in adulthood, it is thus far impossible to perturb precise neurotransmitters, cell types, and circuits in humans (Brockway and Crowley, 2020). Animal models of adolescent alcohol exposure (Crowley et al., 2019) and adolescent stress exposure (Lo Iacono and Carola, 2018; Schroeder et al., 2018) provide a robust opportunity to understand the precise signaling cascades and systems involved in these developmental changes – and will likely provide the first evidence for pharmacological intervention. In addition, although several groups have proposed an association between the age at first alcoholic drink and escalated alcohol use in adulthood, others have proposed that the age at first intoxication or first binge-drinking episode is more important in predicting risk of problems related to excessive alcohol use (Morean et al., 2018; Newton-Howes et al., 2019). This important difference highlights that patterns of alcohol use may play a key role in susceptibility to greater neuropsychiatric disorders – a variable that the animal literature is uniquely poised to investigate.

## Animal Studies of Alcohol Exposure

Animal models of adolescent alcohol consumption have allowed for greater control in alcohol administration while investigating the intersection between genetic background, biological sex, and the role of individual populations of neurons and circuits (Crowley et al., 2019). Using animal models, adolescent-specific patterns of alcohol consumption can be modeled with greater control over patterns of consumption and age windows of exposure. Multiple models have supported the general human phenotype of adolescent alcohol exposure leading to increased consumption of alcohol in adulthood. Previous work has shown that while C57Bl/6J mice exposed to Drinking-in-the-Dark (DID), an NIAAA-recognized model of binge drinking, as adolescents (PND 25–45) show escalated alcohol consumption as adults, this did not hold true for DBA/2J mice (Moore et al., 2010). The adolescent data shown by Moore et al. (2010) is consistent with the general belief of C57Bl/6J mice as “high drinkers” and DBA/2J mice as “low drinkers” and importantly, hints at the potential for genetic mechanisms by which some mice escalate drinking in adulthood. However, recent work suggests that DBA/2J mice will consume alcohol when administered intragastrically, and thus likely are taste-avoidant for alcohol, not alcohol-avoidant more generally (Fidler et al., 2011). Importantly, the study by Moore et al. (2010) was one of the first to include males and females. Other studies have continued to investigate the relationship between adolescent alcohol exposure, adulthood alcohol consumption, and sex. Another model of binge drinking during adolescence increased alcohol consumption in adulthood, with the effect primarily driven by higher drinking in females (Strong et al., 2010). Not all work shows a sex-dependent effect, however. Younis et al. (2019) demonstrated that adolescent DID (PND 28–36) increased alcohol consumption in adults (PND 72–80), without sex differences in either adolescent or adulthood drinking levels.

Although the previously discussed results indicate that adolescent alcohol exposure increases alcohol consumption in adulthood in rodents, other studies do not support this relationship. Rats which drank during adolescence did not show increased consumption compared to naïve rats when presented with alcohol in adulthood (Vetter et al., 2007). Importantly, forced exposure (non-choice consumption models) are also used in rodents, and while they lead to more consistency in alcohol exposure, they may produce differing effects. For example, vaporized ethanol exposure during adolescence did not increase voluntary consumption in adults (Jury et al., 2017). It is difficult to tease apart whether a lack of escalation in adulthood drinking seen in these cases is driven by exposure model, species (rats versus mice), overall amount of ethanol, or developmental window.

As seen in humans, adolescent rodents display different consumption patterns compared to adults. Studies continue to demonstrate the importance of including both males and females in alcohol studies, as alterations due to adolescent alcohol may be sex specific. Several studies have shown that female rodents consume more alcohol than males in both adolescence and adulthood (e.g., Amodeo et al., 2018; Szumlinski et al., 2019), but occasionally opposite patterns have been observed (Vetter-O’Hagen et al., 2009). Though sex differences in alcohol drinking have not been universally observed, some studies have found that testosterone and estradiol contribute to levels of alcohol consumption in adults. Testosterone was shown to have an inhibitory effect on drinking (Vetter-O’Hagen et al., 2011). Castration of male rats increased alcohol drinking, while subsequent restoration of testosterone reduced drinking to the level of control males (Vetter-O’Hagen et al., 2011). Estradiol (E2) has contrasting effects, as alcohol intake in ovariectomized females was reduced compared to control females but restored following subcutaneous injections of E2 (Satta et al., 2018). In addition to hormonal contributions to drinking, motivation and affect may also contribute to sex differences in alcohol drinking patterns. Adolescent male rats are more likely to engage in drinking in social contexts (Varlinskaya et al., 2015a). In contrast, adolescent female rats consume more ethanol when isolated (Varlinskaya et al., 2015b). Adolescent females showed low social behavior indicative of social anxiety-like behavior and were more likely to experience the anxiolytic and facilitative effects (such as increase their social interaction) following ethanol intake (Varlinskaya et al., 2015a,b). Importantly, these behavioral phenotypes broadly mimic those seen in humans, with men more likely to engage in drinking for positive reinforcement and women more likely to drink for the negative reinforcing effects [reviewed in Dir et al. (2017)].

In addition to changes in adulthood drinking levels, animal studies have reported long term effects of adolescent alcohol exposure on behaviors related to anxiety, risk taking, and memory. These effects may not present until adulthood and may be dependent on species of rodent, sex of the subjects, alcohol exposure paradigm, and timing of exposure. For example, male rats are vulnerable to developing social anxiety-like behaviors following adolescent alcohol exposure (Varlinskaya et al., 2014, 2017, 2020), and both males and females show similar changes in anxiety-like behavior after adolescent alcohol measured with the elevated plus maze (Varlinskaya et al., 2020). However, intermittent alcohol exposure consisting of voluntary drinking sessions intermixed with forced vaporized ethanol exposure throughout adolescence did not affect anxiety-like behavior measured using the light-dark box and marble burying test when tested in adulthood (Amodeo et al., 2018). Importantly, these differing behavioral tests may capture different behavioral phenotypes on an anxiety-like spectrum, and performance in one test should not necessarily be expected to perfectly predict performance in another, thus further muddying the overall interpretation of the literature.

Varlinskaya et al. (2020) also found that males are uniquely susceptible to developing deficits in behavioral flexibility after chronic intermittent adolescent alcohol via intragastric gavage (2020). This change in behavioral flexibility was replicated in another study which used alcohol vapor exposure throughout the early adolescent period (Gass et al., 2014). Only males were used in this study, limiting the ability to draw comparison between the sexes (Gass et al., 2014). Another study found similar reductions in behavioral flexibility in both male and female C57Bl/6J mice which drank during early adolescence (van Hees et al., 2022). It is possible that different alcohol exposure paradigms may differentially affect behavior – with intragastric gavage and vaporized ethanol both reflecting forced exposure, and drinking reflecting choice consumption. Alcohol-induced alterations in other types of behaviors have been primarily studied in males. Adolescent male rats exposed to alcohol via intragastric gavage showed increased risky behavior in a probability discounting task 1 week into withdrawal (Boutros et al., 2015). However, mice bred to prefer alcohol did not show changes in impulsivity following alcohol exposure during the mid-adolescent period (O’Tousa et al., 2013).

**FIGURE 1:**
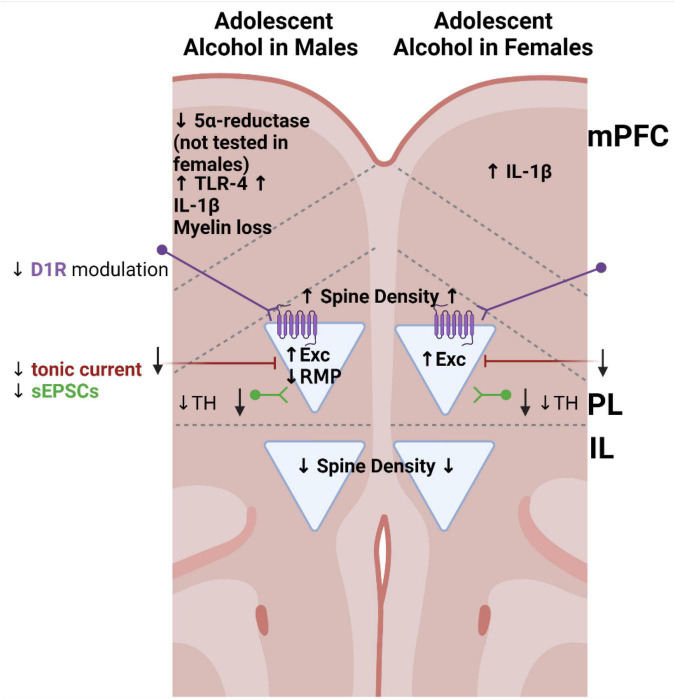
Summary of alterations in the prefrontal cortex identified in rodent models following adolescent alcohol exposure, separated by sex. Triangles represent pyramidal neurons. Arrows indicate the direction of the alteration relative to control animals not exposed to alcohol. Prelimbic pyramidal neurons are more excitable, receive reduced excitatory synaptic input and reduced tonic current, and show increased spine density in both sexes following adolescent alcohol. D1R, dopamine receptor 1; D1R GPCR illustrated in purple; Exc, intrinsic excitability; IL, infralimbic cortex; IL-1β, interleukin 1 beta; mPFC, indicates changes tested throughout the medial prefrontal cortex; PL, prelimbic cortex; sEPSCs, spontaneous excitatory postsynaptic currents; RMP, resting membrane potential; TH, tyrosine hydroxylase; TLR-4, toll-like receptor 4.

Corresponding with persistent changes in behaviors, adolescent alcohol induces alterations in maturing brain regions, including the PFC. Major changes to the PFC induced by adolescent alcohol are summarized in Figure 1. Changes in PFC connectivity have been reported, as have alterations in neuronal structure and function. Adolescent alcohol exposure interferes with several of the maturational processes described above. One important developmental process which occurs during adolescence is the refinement of connections between late-maturing regions like the PFC and already-matured subcortical regions. Ongoing development of frontostriatal connections due to this relatively late maturation of the PFC is hypothesized to contribute to these adolescent-typical behaviors of sensation-seeking and risk-taking (Steinberg, 2008; Shulman et al., 2016). Intermittent exposure to alcohol during adolescence reduces intra-PFC resting-state connectivity and functional connectivity between the PFC and structures in the striatum (Broadwater et al., 2018). The frontal lobe undergoes continued expansion even into late adulthood, as PFC thickness was increased at PND 220 compared to PND 80 (Vetreno et al., 2017). However, rats exposed to alcohol intermittently throughout adolescence (PND 22–55) via intragastric gavage exhibited thinning of the PFC at the younger adult timepoint (PND 80). Importantly, this study only investigated changes in male rats, while neuroimaging studies in human adolescents with a history of binge drinking have shown sex-specific alterations in cortical thinning (de Bellis et al., 2005; Medina et al., 2008; Squeglia et al., 2012). These results show how adolescent alcohol interferes with structural maturation of the PFC and suggest a greater need for in-depth assessment of sex-specific alcohol-induced changes in cortical maturation and connectivity.

At the cell-type specific level, much work assessing changes in PFC neuronal function following adolescent alcohol has focused on excitatory glutamatergic pyramidal neurons, where several studies describe persistent electrophysiological and morphological changes in these output neurons after adolescent alcohol. It has been proposed that adolescent alcohol impairs the development of several neuronal properties, inducing these lasting deficits and possibly contributing to previously discussed behavioral changes in adults exposed to alcohol during adolescence. Pyramidal neurons in layer 5 of the prelimbic (PL) cortical region of the PFC show increased intrinsic excitability following adolescent alcohol exposure, with this effect persisting into adulthood, several weeks after alcohol exposure ended (Salling et al., 2018; Galaj et al., 2020). This effect was specific to alcohol exposure during adolescence (beginning at approximately PND 28), as alcohol exposure in young-adult mice (beginning at PND 70) demonstrated decreased pyramidal neuron intrinsic excitability (Galaj et al., 2020). Alterations in excitability were not seen until 21 days after cessation of alcohol exposure – highlighting the importance of investigating protracted timepoints. In addition, excitatory input onto layer 5 pyramidal neurons was reduced following adolescent alcohol exposure, suggesting that larger network changes occur following alcohol exposure, in addition to alterations in intrinsic excitability (Galaj et al., 2020).

Salling et al. (2018) found that voluntary alcohol consumption during adolescence (PND 30–60) interfered with the typical development of pyramidal neuronal membrane properties including resting membrane potential and voltage sag following injections of hyperpolarizing current. Although PL cortical pyramidal neurons become more depolarized during adolescence, this change did not occur following alcohol exposure where pyramidal neurons remained hyperpolarized (Salling et al., 2018). The hyperpolarization-activated cation (Ih) current was also reduced following adolescent drinking, likely being a causal mechanism for the changes in intrinsic excitability (Salling et al., 2018). Further work from this group showed a role for a hyperpolarization-activated cyclic nucleotide-gated channel (HCN1) in alcohol preference in male adolescent mice (Salling and Harrison, 2020), where they demonstrated HCN1 knockout mice consumed more 20% alcohol in an intermittent voluntary consumption paradigm. This finding has not yet been investigated in females, although female rodents show higher levels of HCN1 expression in PFC Martinotti neurons, hinting at another key interaction between adolescent alcohol consumption and biological sex (Hughes et al., 2020).

Throughout adolescence, expression of a tonic GABA current increases in PL cortical layer 5 pyramidal neurons (Centanni et al., 2017). Although the amplitude of the current is consistent throughout development, the percentage of pyramidal neurons showing this tonic current increases during the adolescent period. As expression of the tonic GABA current increases, so does the contribution of delta-subunit containing GABA_*A*_ receptors. However, this work by Centanni et al. (2017) found that binge-like intermittent alcohol vapor exposure during adolescence blocks the development of this current and reduces its amplitude. This reduction appeared within days of adolescent alcohol and persisted into adulthood in both males and females. Adolescent alcohol also attenuated the effects of allopregnanolone, a neuroactive steroid which has been shown to increase this tonic current (Stell et al., 2003). The authors hypothesize that adolescent alcohol alters the performance of delta subunit-containing GABA_*A*_ receptors without affecting expression of the delta-subunit (Centanni et al., 2017). This suggests a greater need for investigation of the alcohol-induced alterations in receptor function.

Adolescent alcohol prevents the typical development of dopamine modulation of pyramidal cell firing in the PL cortex (Trantham-Davidson et al., 2017), another PFC system known to undergo developmental maturation. During adolescence, the number of pyramidal neurons which show D1R and D2R modulation of activity increases. However, following adolescent alcohol exposure, stimulating D1Rs no longer changed evoked firing of pyramidal neurons, indicating a loss of this development (Trantham-Davidson et al., 2017). Interestingly, these changes were not due to changes in D1R expression in these neurons (Trantham-Davidson et al., 2017), and there was no change in D2R-modulated firing. In addition, electrophysiological alterations were reported along with reduced PL cortical expression of tyrosine hydroxylase, a marker for dopaminergic neurons, following adolescent alcohol exposure (Trantham-Davidson et al., 2017). These findings indicate that not only is development of dopaminergic inputs into the PFC altered by adolescent alcohol, but that the functioning of corresponding receptors may change as well, and that the alterations have consequences for the firing of both pyramidal and non-pyramidal neurons. This highlights the need for in-depth investigation of entire neurotransmitter systems.

Morphological changes to pyramidal neurons, including alterations in dendritic spines, have been proposed to underlie age-specific differences in the effects of adolescent alcohol (Galaj et al., 2020). Indeed, adolescent alcohol increases the prevalence of thin dendritic spines, which are hypothesized to have fewer receptors than other non-thin dendritic spines, as well as increase overall dendritic spine density on PL pyramidal neurons (Trantham-Davidson et al., 2017; Galaj et al., 2020). However, this effect does not seem to generalize to other PFC regions. Exposure to alcohol vapor during adolescence reduced the density of dendritic spines in another region of the PFC, the infralimbic cortex, with average dendritic spine width increasing after adolescent alcohol (Jury et al., 2017).

The cholinergic system is also vulnerable to adolescent alcohol use. Intermittent alcohol exposure via intragastric gavage throughout adolescence reduced cholinergic markers in the orbitofrontal cortex (OFC) of male and female Sprague Dawley rats (Kipp et al., 2021) and in the basal forebrain male in Wistar rats (Boutros et al., 2015). Dendritic branching complexity was altered in the OFC, though the location of compromised branching was different in alcohol-exposed males and females (Kipp et al., 2021). Cholinergic efflux in the OFC, but not the hippocampus, during behavioral tasks is reduced following adolescent intermittent alcohol (Kipp et al., 2021).

Another critical developmental change during adolescence which is susceptible to alcohol use is the myelination of neurons within the PFC. Voluntary alcohol consumption reduced myelination density in the anterior cingulate cortex of male Wistar rats (Tavares et al., 2019). Although overall density of myelinated fibers was unaltered in females, adolescent alcohol consumption induced microstructural changes in female rats, including a shift toward smaller nodes of ranvier (Tavares et al., 2019). Based on their findings, the authors propose that adolescent alcohol alters myelin density by inducing demyelination followed by remyelination, with males being more vulnerable to these consequences. Similarly, Rice et al. (2019) found that adolescent alcohol reduced myelin basic protein (MBP) density in the medial PFC in adolescent male C57BL/6J mice, but this deficit was restored by adulthood. Immediately following adolescent alcohol, there was an increase in the expression of degraded MBP in the medial PFC (Rice et al., 2019). This result seems to match the demyelination-remyelination hypothesis proposed by Tavares et al. (2019). The increased vulnerability of PFC myelin in males may be due to sex differences in expression of inflammatory markers following adolescent drinking (Silva-Gotay et al., 2021). Expression of some pro-inflammatory genes, including interleukin 1-beta, increases in the PFC of both males and females after adolescent alcohol, while expression of the gene encoding Toll-like receptor 4 (TLR4) is increased in the PFC only in males (Silva-Gotay et al., 2021). Because TLR4 has been implicated in alcohol-induced myelin loss (Montesinos et al., 2015), Silva-Gotay et al. (2021) argue that this sex difference in inflammatory gene expression may contribute to the increased sensitivity to adolescent alcohol in males. Overall, these results indicate that myelination during adolescence is susceptible to alcohol exposure in both males and females. Males may be more vulnerable to these changes in myelination; however, more studies investigating the mechanism of myelin loss in both males and females are needed. Given the devastating effects of myelination-related neurodegenerative disorders in adulthood, a thorough understanding of how adolescent alcohol may contribute to this phenotype is absolutely critical to preventing and intervention of these diseases in aging populations.

Neuroactive steroids in the PFC are targets of alcohol and have been identified as potential treatment options to reduce excessive drinking. Male Wistar rats exposed to ethanol during adolescence showed increased mRNA and protein levels of 5 alpha-reductase in the PFC (Sánchez et al., 2014). The magnitude of change was highest in rats exposed to ethanol on a chronic intermittent schedule (Sánchez et al., 2014). Administration of neuroactive steroids has reduced voluntary alcohol consumption in male rats exposed to ethanol during adolescence (Gurkovskaya et al., 2009). Because of this demonstrated effect, neuroactive steroids have emerged as a potential treatment strategy for AUD (Morrow et al., 2020); however, studies testing the efficacy of neuroactive steroids across sex and broader age ranges are limited.

Together, the human and animal literature both demonstrate that adolescents are especially at risk of initiating alcohol use and that alcohol can interfere with PFC development during this critical stage. Studies using rodents have found major neurobiological changes following alcohol at the structural and cellular level, but factors including sex, species, alcohol exposure paradigm, and timing of exposure may influence results. Rates of alcohol use among adolescents remain high, making it prudent to understand persistent changes of adolescent alcohol consumption.

## Human Studies of Adolescent Stress

While adolescence is a time of great physiological and psychological change, this change is often accompanied by varying levels of stress. Chronic exposure to stress in adolescence is associated with alterations in cortical structure, and an increased risk of neuropsychiatric illnesses, including AUD (Watt et al., 2017). Many neuropsychiatric illnesses begin to present during adolescence, possibly because the PFC, implicated in several such disorders, is especially vulnerable to factors including stress while it undergoes the aforementioned maturational changes (Paus et al., 2008). Ongoing stressors resulting from the COVID-19 pandemic have increased the prevalence of anxiety and depression symptoms among adolescents worldwide (Racine et al., 2021). Sex differences in the prevalence and progression of neuropsychiatric illnesses such as MDD (Schuch et al., 2014), PTSD (Olff, 2017), and anxiety disorders (McLean et al., 2011) and other disorders indicate potential biological differences in stress reactivity between men and women.

Engagement of the hypothalamic-pituitary-adrenal (HPA) axis is a classical response to stress, resulting in altered circulating cortisol levels as a response, although the directionality of a possible sex difference in response to more chronic stress is unclear as the literature reports conflicting findings (for review, see Bangasser and Valentino, 2014). Continued higher cortisol levels are often seen in patients with MDD, and lower cortisol levels are seen in people with PTSD (Bangasser and Valentino, 2014). Studies report women suffering from depression tend to have higher cortisol levels than men with depression (Young and Altemus, 2004), and women with PTSD show lower cortisol levels than women without PTSD, but the effect is not seen in men (Freidenberg et al., 2010; Bangasser and Valentino, 2014). Another way the HPA axis is implicated is through the release of corticotropin-releasing factor (CRF), increased levels of which are seen following stress (Sautter et al., 2003; Bangasser and Valentino, 2014), and increased sensitivity to CRF is noted in women during puberty (Stroud et al., 2011), implicating a possible mediating role for ovarian hormones in stress sensitivity during and post adolescence. Of note, CRF is also released by CRF containing neurons throughout the brain and not solely regulated by the HPA axis (Kash et al., 2015; Brockway and Crowley, 2020).

Other alterations to brain maturation seen due to stress exposure include myelination deficits (Shaw et al., 2020) and synaptic pruning leading to decreases in cortical gray matter (Watt et al., 2017; Shaw et al., 2020). Myelination of the central nervous system continues into adulthood (Vetreno et al., 2017), and is highly sensitive to stress, with the literature reporting the implication of myelin deficits in neuropsychiatric illnesses via a multitude of neuroimaging-based white matter studies (for review, see Tae et al., 2018). White matter structure was shown to be decreased after adolescent stress using male subjects from the Avon Longitudinal Study of Parents and Children, specifically in the corpus callosum (Jensen et al., 2018).

As highlighted above, the adolescent brain undergoes significant synaptic pruning, with synaptic density within the PFC peaking at the onset of puberty and declining rapidly before stabilizing at the end of adolescence (Shaw et al., 2020). While unconfirmed in humans, studies show decreased dendritic spine density and branching in pyramidal neurons in layer II/III of adult rodent PFC after exposure to chronic stress (for review, see Romeo, 2017). As with much of the human literature, it is difficult to tease apart cellular level findings after stress, but the human literature is plentiful with neuroimaging studies post-stress to provide some mechanistic insight. Watt et al. (2017) hypothesize that the disruption of circuitry of the PFC caused by adolescent stress leads to impaired reward processing, leading in turn, to struggles with impulse control and a higher susceptibility to substance use disorders and neuropsychiatric illnesses. Findings of a neuroimaging study of 20-year-old men who experienced chronic stress between 15 and 18 years of age showed decreased PFC activation in response to reward anticipation (Casement et al., 2013), supporting the hypothesis of Watt et al. (2017). A study of young women who were child sexual abuse victims (ages 14–16 at time of abuse, and 17–19 at time of study) showed decreased frontal lobe gray matter (Andersen et al., 2008), indicating a potential long-term stress-induced adaptation in cortical development. An adolescent-specific stress effect was seen by Baker et al. (2013) when imaging populations of adults (ages 30–40) who had experienced early life stress. Interestingly, individuals who had faced chronic stress between the ages of 8–17 showed decreased gray matter in the anterior cingulate cortex and insular cortex within the frontal lobe, but no such decrease in gray matter was seen in individuals who had faced chronic stress before age 8 (Baker et al., 2013). Combined, these studies give support to the possibility of enhanced cortical vulnerability to stress during adolescence. Importantly however, adolescent stress in humans is often complicated by interactions between variables that synergize with stress, including socioeconomic status, food and housing security, and education.

## Relationship Between Stress and Alcohol Use

The AUD shows high co-morbidity with other neuropsychiatric illnesses, including depression, bipolar disorder, and borderline and antisocial personality disorders (Grant et al., 2015). The presence of an AUD has also been associated with heightened severity of these co-morbid disorders (Helle et al., 2020). As previously mentioned, there is an complex relationship between anxiety and alcohol consumption. Anxiety disorders and AUD are often co-morbid (Grant et al., 2004), but there have been conflicting findings indicating the exact characteristics and mechanisms of this relationship (e.g., see Dyer et al., 2019). The uncertainty around this mechanism or directionality of these comorbidities extends to the adolescent literature as well. Cloutier et al. (2019) found that adolescent females with higher baseline social anxiety levels are more likely to consume their first alcoholic beverage within a year of exposure to an acute social stressor. However, anxiety symptoms alone do not necessarily lead to an increased urge to drink immediately in non-alcohol naïve adolescents (Blumenthal et al., 2015), indicating that there are other factors at play in the relationship between adolescent alcohol consumption and anxiety that require further study (with access to alcohol likely being relevant). The same must be said of other common comorbidities of AUD, including MDD and conduct disorder due to the tendency to use alcohol as a coping method for psychological distress (Zaso et al., 2021) and as a negative reinforcer among late-stage adolescents (Obasi et al., 2016). Obasi et al. (2016) surveyed a population of undergraduate college students, examining the relationship between psychological stress and alcohol use, and report the relationship between individuals with problem drinking (as assessed by the Alcohol Use Disorders Identification Test) positively correlates with poor emotional coping skills, depression, anxiety, and positive alcohol related expectancies. A model fit was able to predict that the relationship between depression and anxiety and alcohol use is mediated by a chain of negative affect regulation and positive alcohol expectancies (Obasi et al., 2016). Importantly, work like this can help to tie together the correlation and potential causality between overlapping neuropsychiatric disorders.

## Animal Studies of Adolescent Stress

**FIGURE 2:**
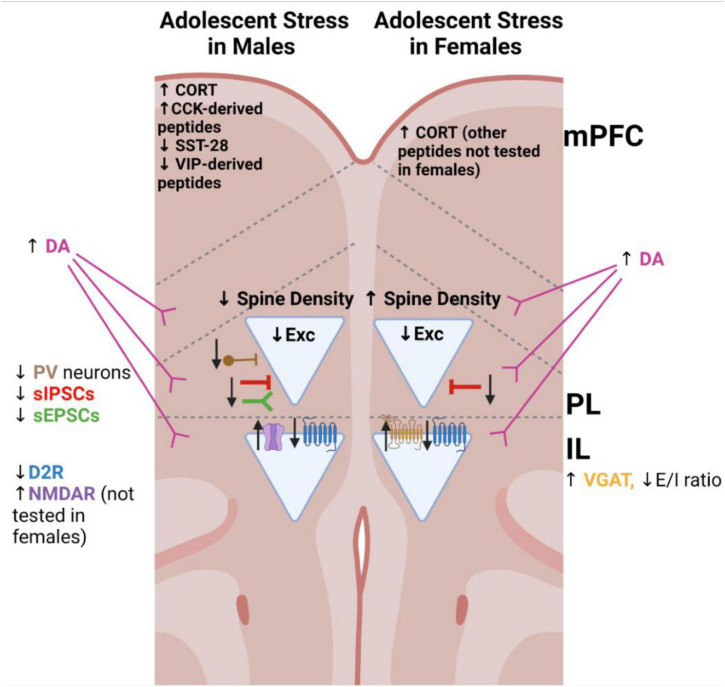
Schematic of major changes to the medial prefrontal cortex identified in rodent models of adolescent stress. Triangles indicate pyramidal neurons. Arrows represent the direction of the alteration, relative to non-stressed controls of the same sex. CCK, cholecystokinin; CORT, corticosterone; D2R, dopamine receptor 2; D2R GPCR illustrated in blue; DA, dopamine; E/I ratio, excitatory/inhibitory balance; IL, infralimbic cortex; mPFC, medial prefrontal cortex (indicates alterations tested throughout this region); NMDAR, *N*-methyl-D-aspartate receptor; PL, prelimbic cortex; PV, parvalbumin-expressing; sIPSCs, spontaneous inhibitory postsynaptic currents; sEPSCs, spontaneous excitatory postsynaptic currents; SST-28, somatostatin-28 peptide; VGAT, vesicular GABA transporter 1; VIP, vasoactive intestinal peptide.

As with alcohol consumption, exposure to stress during adolescence can impair typical maturation of the brain and lead to persistent changes in behavior and neurobiology. Animal models present a unique opportunity to not only understand causality between adolescent stress exposure and related disorders, but also to understand when unique periods of vulnerability occur, and how these interact with cell- and circuit- level specific changes in the brain. Various types of stressors have been used, including social defeat, social isolation, physical stress, and variable stress paradigms. Neurobiological changes in the PFC following adolescent stress are outlined in Figure 2.

Unsurprisingly, the brain is uniquely susceptible to stress during adolescent development. Several studies have reported unique behavioral changes in animals which underwent stress during adolescence compared to animals which underwent stress in adulthood. Exposure to an elevated-platform stressor in early adolescence, but not adulthood, impaired reactivity to an acute stressor presented later in life (Avital and Richter-Levin, 2005). When male and female mice were exposed to a footshock stress in either mid-adolescence (PND 36–37) or adulthood (PND 72–77), sex- and age-specific effects presented. In this study, females demonstrated an increased startle response after either adolescent or adult stress, while adult males exposed to stress showed a reduction in startle response compared to non-stressed males (Chester et al., 2008). Other studies have found increased anxiety-like behavior following adolescent stress (Luo et al., 2014; Page and Coutellier, 2018). Female rats are more vulnerable to lasting anxiogenic effects of adolescent social stress than males (McCormick et al., 2008), although other studies show persistent increases in anxiety-like behavior in stressed animals of both sexes compared to unstressed controls (Page and Coutellier, 2018). Stressed female Listar hooded rats exhibited more nose-poking behavior during a delay discounting task, interpreted as compulsive-like behavior (Brydges et al., 2015). Adolescent stress induces lasting effects, most notably increasing anxiety-like behavior in adult rodents, with evidence suggesting that females may be more vulnerable than males.

Animal models of adolescent stress have also been shown to influence alcohol consumption in adulthood, with evidence of sex-specific effects. Exposure to chronic variable social stress (CVSS) during adolescence (PND 25–59) significantly increased adulthood alcohol consumption in the DID model of binge drinking (Caruso et al., 2018). An effect of sex on alcohol consumption was not reported, but stressed females drank to higher blood alcohol levels compared to non-stressed females (Caruso et al., 2018).

Post-weaning social isolation has been used to model adolescent stress in rats and mice and to investigate the relationship between adolescent stress and alcohol intake. Social isolation beginning at weaning (PND 21) led to increased drinking in adult mice (Lopez et al., 2011; Lopez and Laber, 2015) and rats (Hall et al., 1998). In contrast, adulthood social isolation for the same length of time did not influence levels of alcohol intake (Lopez et al., 2011). Drinking levels following adolescent social isolation were similar to those following chronic variable stress (Lopez et al., 2011). Housing in an enriched environment could reduce alcohol drinking levels in mice following adolescent social isolation to drinking levels of group-housed mice (Lopez and Laber, 2015). However, not all studies have found this relationship, indicating that other factors may affect the relationship between stress and alcohol intake. Both studies by Lopez et al. (2011) used a limited-access model, with mice only having access to alcohol for 2 h each day. In contrast to these results, social isolation upon weaning increased adulthood alcohol consumption and intake measured by 2-bottle choice in males only (Advani et al., 2007). Moriya et al. (2015) did not find any change in alcohol drinking following social isolation in C57BL/6J mice. This literature suggests a complex relationship between adolescent social isolation and adulthood alcohol consumption.

Mild foot shock and restraint stress have both been used to model mild physical stress in adolescence. A mild foot shock during adolescence but not adulthood led to higher levels of alcohol consumption in a strain of alcohol-preferring mice (Chester et al., 2008). Exposure to a restraint stress did not alter alcohol consumption in a continuous access paradigm in mice which began drinking during adolescence (Tambour et al., 2008). These mice had several weeks of drinking experience before the stressor occurred (Tambour et al., 2008), so these mice may have been habituated to the effects of alcohol which may have interacted with any changes following stress. Another study showed that restraint stress during early adolescence altered alcohol consumption in an intermittent-access paradigm in a sex- and age-specific manner (Wille-Bille et al., 2017). Adolescent males and adult females decreased drinking following stress, while adolescent females increased alcohol consumption. Other factors which may mediate the relationship between adolescent stress and alcohol consumption may include the experimental stress paradigm, method to assess subsequent alcohol consumption, and time between adolescent stress and measurement of adult alcohol consumption.

Adolescent stress imposes neurobiological changes which underlie these behavioral consequences by impairing several critical maturational changes. These major changes within the PFC following adolescent stress are visualized in Figure 2. Supporting this, lesions of the PL region of the PFC increased vulnerability to adolescent footshock and restraint stress (Gomes and Grace, 2017). Specific targets of adolescent stress in the PFC may include neurotransmitter systems, neuronal populations, peptides, and hormones, as well as developmental changes affecting these target systems.

In the PFC, the impacts of adolescent stress on development of the GABAergic system have been characterized, with research investigating several subpopulations of GABAergic neurons. Development of the GABAergic system is necessary for proper cortical inhibitory/excitatory balance in adulthood, and impairments in this system have consequences for emotional development (Page and Coutellier, 2019). This development can be altered by adolescent stress paradigms in a sex-specific manner. For example, adolescent stress reduces the number of PV neurons in the PL cortex only in male mice [Page and Coutellier, 2018; reviewed in Woodward and Coutellier (2021)]. However, the effects of stress throughout the lifespan on other populations of GABAergic neurons have not been studied as extensively. Other GABAergic populations including SST- and VIP- expressing neurons are potential targets for specific changes in the GABAergic system following adolescent stress. In animal models, SST-expressing neurons have roles in depressive-like phenotypes (Fuchs et al., 2017), substance misuse (Dao et al., 2020; Crowley and Joffe, 2021), and encoding of fear memories (Cummings and Clem, 2020) in adult mice. Although membrane properties of SST-expressing neurons in the PFC are stable by adolescence (Pan et al., 2016; Koppensteiner et al., 2019), SST neuron-mediated inhibitory transmission onto pyramidal neurons in the infralimbic cortex is reduced following fear conditioning in adolescent mice (Koppensteiner et al., 2019). This effect of fear conditioning was not observed in pre-adolescent or adult male mice (Koppensteiner et al., 2019). Future research should continue to investigate these other GABAergic populations in both males and females after adolescent stress.

Adolescent stress altered the expression of several neuropeptides in the PFC of male Wistar Han rats. After a stress paradigm of unpredictable exposures to fox odor or an elevated platform from PND 28–42, levels of SST-28 were reduced (Li et al., 2018). SST has been implicated in human neuropsychiatric illnesses (Brockway and Crowley, 2020; Robinson and Thiele, 2020) and is an interesting target for future research in adolescent stress. Other stress-affected neuropeptides in the PFC include cholecystokinin (CCK) and VIP precursors (Li et al., 2018). Stress hormones are also persistently affected by adolescent stress. Following prolonged post-weaning social isolation, corticosterone levels increased in both males and females (Pisu et al., 2016). This finding has important implications for the alcohol experiments discussed above, such as those using the DID model, which require social isolation. Although acute footshock stress in adolescence slightly increased baseline corticosterone levels in adulthood, exposure to this stressor did not influence corticosterone response to an acute stress challenge in adulthood (Lovelock and Deak, 2017, 2020).

Neurotransmitter systems such as the dopaminergic system and its alteration by adolescent social stress have undergone in-depth investigation. For example, Watt et al. (2014) exposed male Sprague-Dawley rats to social defeat stress beginning in either mid-adolescence (PND 35) or adulthood (PND 70). Several weeks later, dopaminergic activity in the medial PFC (measured by levels of dopamine and the dopamine metabolite 3,4-dihydroxyphenylacetic acid) was reduced in rats which underwent adolescent stress. In rats which were not stressed until adulthood, levels of dopaminergic activity were increased at the same timepoint after stress (Watt et al., 2014). Others have found that stress beginning in early adolescence, however, corresponded to an increase in medial PFC dopamine levels (Luo et al., 2014). This suggests that stressor timing during adolescence can influence the direction of dopamine alterations. Supporting this, immunostaining for tyrosine hydroxylase increased in layer I of the PL cortex of male and female rats stressed from PND 27–29, a relatively short period of time immediately surrounding the start of adolescence (Harris et al., 2022). Exposure to a predator odor during adolescence reduced D2R expression in the medial PFC in adulthood (Wright et al., 2008), perhaps indicating compensation for changes in dopamine levels following adolescent stress. Importantly, this mimics the effects seen with alcohol in that both neurotransmitter ligands and receptors tend to be affected by developmental perturbations.

During adolescence, inputs onto pyramidal neurons in regions including the PFC continue to mature; however, this maturation is disrupted by adolescent stress. Social stress during adolescence alters inhibitory transmission onto layer II/III pyramidal neurons in the adult medial PFC (Caruso et al., 2018; Xu et al., 2021). Glutamatergic transmission onto PL cortical pyramidal neurons is reduced following adolescent stress in males (Urban and Valentino, 2017; Caruso et al., 2018). Properties of pyramidal neurons are also altered by adolescent stress. While stress beginning in early adolescence did not alter pyramidal neuron intrinsic excitability, stress beginning in mid-adolescence resulted in reduced intrinsic excitability of PL cortical pyramidal neurons in males and females (Urban and Valentino, 2017). These results demonstrate the importance of stress timing in neurobiological outcomes and indicate that mid-adolescence may be a particularly sensitive period for changes in the PFC following stress. Along with electrophysiological changes, adolescent stress induces sex-specific morphological changes in PFC pyramidal neurons. After an unpredictable stress paradigm, dendritic spine density increased in stressed females but decreased in stressed males (Urban et al., 2019). The balance of the excitatory/inhibitory synaptic markers vesicular glutamate transporter 1 and vesicular GABA transporter were reduced in stressed females, indicating potential alterations of the excitatory/inhibitory balance (Bueno-Fernandez et al., 2021).

Adolescent stress impairs development of NMDA receptor expression in the PFC. Stressed male rodents show increased levels of NMDA receptor expression in the infralimbic region of the PFC (Novick et al., 2016). Expression of the NMDA receptor NR2B subunit is increased in the PFC of males but not females after early-adolescent stress (Page and Coutellier, 2018). NMDA receptor subunit expression has been shown to stabilize before adolescence (Ueda et al., 2015), so adolescent stress may interfere with or override this stabilization. Together, these findings indicate that exposure to a variety of stressors during adolescence can cause persistent changes in behavior and corresponding changes in the PFC, with factors including sex, stress paradigm, and timing of exposure mediating these changes.

## Discussion

Human and animal studies indicate that the adolescent period is particularly vulnerable to alcohol- and stress-induced changes in brain development because both alcohol and stress exposure interfere with necessary maturational processes naturally occurring in critical brain regions such as the PFC during this time. Stress and alcohol use are common among adolescents, and the ongoing COVID-19 pandemic is likely to exacerbate both of these phenomena. The American Psychological Association’s 2020 Stress in America survey found that over 40% of teenagers aged 13–17 report that their stress levels had increased over the previous year, due to the pandemic’s disruptions to their daily lives and uncertainty about their future (Stress In America, 2020). Adolescents and young adults aged 18–23 reported higher levels of stress, on average, than adults over the age of 24 (Stress In America, 2020). Alarmingly, alcohol use among adolescents has plateaued in recent years after several decades of decline – which may be related to the increased rates of stress caused by the COVID-19 pandemic (CDC, 2020). Together, the prevalence of alcohol use and stress-related pathology during adolescence, and the changing rates of both of these phenomena during the current pandemic, highlight the importance of understanding the neurobiological consequences, both short- and long-term, of adolescent stress exposure and alcohol consumption.

Although the literature discussed above indicates a variety of behavior and neurobiological characteristics which are sensitive to adolescent alcohol or stress exposure, it is paramount to note the vast variability in standard operating procedures for both preclinical stress and alcohol paradigms, which likely are a key driver in the discrepancies in effects seen in the literature. For example, though it has been argued that alcohol exposure during adolescence increases alcohol consumption in adulthood, not all studies have shown this relationship. Other factors which might mediate the effects of adolescent alcohol and stress include animal strain, timing of the experiment during adolescence, and duration of the stressor. In addition, new research indicates variables as seemingly innocuous as lab diet may influence alcohol consumption – making it particularly difficult to rectify differences in the literature (Maphis et al., 2022). Because the variety of paradigms used to induce stress or alcohol exposure can be used to model different consequences of these perturbations (e.g., see McCormick et al., 2017; Crowley et al., 2019), more standardized protocols across alcohol and stress exposure paradigms may reduce some of this variability. However, the variability in outcomes does not diminish the overall finding that the adolescent period is particularly vulnerable to lasting consequences of alcohol and stress.

Despite the National Institute of Health 2016 mandate to include sex as a biological variable (SABV) (Consideration of Sex as a Biological Variable in NIH-funded Research, 2016), there remains a large gap in the literature around the effects of alcohol exposure and stress on cortical neurocircuitry in females. Addressing this gap will allow for a fuller and more robust understanding of the development of adolescent cortical neurocircuitry into adulthood. Although many of the studies reviewed here found that sex influences how the brain changes in response to alcohol, existing treatment for AUD and neuropsychiatric illnesses in general tend to be standardized for both sexes (Bekker and van Mens-Verhulst, 2007; Holzhauer et al., 2019). In fact, the current understanding of the efficacy of several AUD pharmacotherapies is based mainly on studied responses in males. Some analyses have found that clinical trials for several pharmacotherapies for AUD and alcohol withdrawal, including disulfiram, benzodiazepines, and anticonvulsants, did not include a sufficient number of female participants (Agabio et al., 2016). Although AUD is diagnosed more frequently in men, alcohol use is increasing in women (White, 2019), making it crucial to evaluate pharmacotherapies for AUD in both sexes. Among adolescents, the prevalence of alcohol use is equal in boys and girls, probably due to a sharper decline in alcohol use among adolescent boys (White, 2019), suggesting that the gap in AUD prevalence may continue to close.

While the factor of sex in human studies of alcohol use and stress is important, considering gender identity is crucial to our understanding of alcohol use throughout the lifespan. Although newer studies are beginning to include gender identity as a factor in studies of stress and drinking, much of our understanding of adolescent stress and alcohol use only considers biological sex as a binary variable. Transgender and gender non-conforming adolescents are at increased risk for using alcohol and other drugs, and they are victims of disproportionate amounts of stress as a result of bullying and abuse from peers and family (Reisner et al., 2015; de Pedro et al., 2017). Understanding the lifelong consequences of this unique stress burden faced by transgender and gender non-conforming adolescents is critically important for attempts to reduce alcohol use among these vulnerable populations, as well as for providing targeted interventions and care.

An additional factor for consideration in human studies of alcohol use is the pattern of alcohol consumption. Age of alcohol use onset or age of first intoxication are often primary outcomes of studies; however, this simplifies alcohol use to a binary variable. Monitoring frequency or amount of drinking, which are variables often included in animal studies, will strengthen future studies of human adolescent neurobiology. Importantly, assessment of some of these variables is likely limited due to the retrospective nature of human data science; ongoing studies will likely capture adolescent drinking in far greater detail along these variables. Adolescence is a critical developmental period characterized by behavioral, physical, and neurobiological maturation. Development of the PFC involves a variety of changes, such as myelination, synaptic pruning, and refinement of several key neurotransmitter systems. These maturational changes are susceptible to exposure to alcohol or stress during this period, with behavioral and structural changes often becoming permanent. Promising targets for future research may include populations of GABAergic neurons, as alcohol and stress both impact this system (Morrow et al., 2020; Woodward and Coutellier, 2021). Further investigation into behavioral effects of adolescent alcohol or stress may uncover potential factors influencing individual sensitivity to these insults, as there is often variability in outcomes in human studies. Finally, research should continue to incorporate participants of both sexes to generate a complete understanding of these effects. Because of high reported rates of stress and alcohol use in adolescents, elucidating the age- and sex-specific effects of these insults will likely improve the quality of therapeutic options for AUD and other neuropsychiatric conditions.

## Data Availability Statement

The original contribution spresented in the study are included in the article/supplementary material, further inquiries can be directed to the corresponding author.

## Author Contributions

AS made the figures. All authors contributed to the literature review, writing, and editing of the manuscript.

## Conflict of Interest

The authors declare that the research was conducted in the absence of any commercial or financial relationships that could be construed as a potential conflict of interest.

## Publisher’s Note

All claims expressed in this article are solely those of the authors and do not necessarily represent those of their affiliated organizations, or those of the publisher, the editors and the reviewers. Any product that may be evaluated in this article, or claim that may be made by its manufacturer, is not guaranteed or endorsed by the publisher.
